# Supplementary material for: Deciphering cancer genomes with GenomeSpy: a grammar-based visualization toolkit
Source: Gigascience. 2024 Aug 5;13:giae040. doi: 10.1093/gigascience/giae040 (PMC11299109; doi:10.1093/gigascience/giae040)
Supplement: giae040_Response_to_Reviewer_Comments_Original_Submission [file giae040_response_to_reviewer_comments_original_submission.pdf]

Author's Response To Reviewer Comments

We thank the reviewers for their insightful and constructive comments. We have revised the manuscript according to the comments and this has further improved the manuscript.

Summary of the major modifications

In this second revised version, we:
• Refined the introduction and discussion to emphasize the challenges researchers face in creating customized visualizations that are interactive and to detail how GenomeSpy's JSON-based grammar paves the way for future advancements.
• Emphasized GenomeSpy's customizable data transformation pipeline and its capability to eliminate the requirement for specialized server-side infrastructure.
• Enhanced the DECIDER visualization's G score calculation by introducing user-configurable thresholds, showcasing GenomeSpy's new parameterization feature.
• Included a link to the DECIDER visualization specification and comprehensive documentation, demonstrating the process of constructing such visualizations with GenomeSpy.
• Omitted the COSMIC Cancer Gene Census track in the DECIDER visualization due to its restricted license and lack of usage in our analyses.

Detailed responses to comments by reviewers

Reviewer 1

"In this manuscript, the authors present Genome Spy, a visualization toolkit geared toward the rapid and interactive exploration of genomic features. They demonstrate how this tool can help investigators explore a large cohort of 753 ovarian cancers sequenced by whole-genome sequencing (WGS). By using the tool, they were able to identify outliers in the dataset and refine their diagnosis. The tool is inspired by Vega-lite, a high-level grammar for interactive graphics, and extends it for genomic applications.
The manuscript is clearly written, and the authors provide links to the applications itself, tutorials and examples. I want to commend them for doing this. This is a tool that would nicely complement others and has a specific advantage of using high-performance GPUs that are now common in modern computers.
The only concern that I have is about a couple of claims that may not be fully supported by the data provided:
1. Claim: users can implement new visualization designs easily. While the grammar certainly enables the users to define new designs, I do not think that this is necessarily easy, as the authors themselves recognize in the discussion section when they suggest providing templates to reduce the learning curve. Indeed, the example in Figure 2 is still quite verbose and would need some time for anyone to understand the syntax and the style. The playground web application facilitates testing it, though."

While we agree that the meaning of "easy" is subjective, we point out that using and designing new visualizations is easier when compared to existing tools used in interactive genome visualization. Some programming or specification skills are needed when designing new visualizations with GenomeSpy. In the revised version, we have addressed the ease of use and learning curve by providing a downloadable version of the DECIDER GenomeSpy visualization with documentation and example data and by addressing these concerns by revising the text. The downloadable specification with documentation (https://github.com/HautaniemiLab/genomespy-paper-2024-spec) provides the users with a starting point that allows the visualization to be adapted for their own data.

In the text, we included additional context regarding the relative ease of implementing new visualization designs. In Introduction, we underscore the inherent difficulty most researchers encounter in crafting tailored, especially interactive, visualizations, citing recent literature that corroborates this challenge. In the discussion, we acknowledge the existence of a learning curve with GenomeSpy's grammar-based approach, similar to that of other grammar-based visualization tools. However, we further elaborate that our JSON-based grammar serves as a critical foundation for the development of more intuitive authoring

methodologies for GenomeSpy, anticipated in the future.

"2. Claim: the grammar-based approach allows to be mixed and matched. I did not find any specific example of how to do this. It would have been quite interesting to see the intersection between the DNA representation of structural variants and RNA-seq data (if this is what it means as "mix and match")"

We apologize for unclear writing and have rewritten the concluding paragraph. We now state that the building blocks of GenomeSpy's grammar "can be combined and reconfigured in innovative ways". When it comes to structural variants (SV) and RNA-seq, it is possible to display SVs for multiple samples using GenomeSpy . Expression levels can be shown as segment features mapped onto gene's coordinates, or they can be shown in the metadata heatmap for a limited number of genes. In the DECIDER GenomeSpy visualization, we included multiple data layers: short mutations, copy-numbers and samples specific data such as purities, providing example of combined data types.

(A screenshot illustrating SVs for multiple samples: https://github.com/genome-spy/genome-spy/issues/211)

Reviewer 2

"In this article, the authors introduce GenomeSpy, a grammar-based toolkit for creating customized, interactive visualizations for genomic data analysis. I find the article extremely interesting, and I believe the framework introduced by the authors has broad utility. The website is well-maintained and documented, and I particularly found the examples mentioned in the paper to be useful and informative. The authors chose to present their toolkit by narrating the navigation of a dataset generated in the DECIDER study.
While the narrative makes the utility of the visualizations clear in data interpretation, what is not clear at all is how easy it is to use GenomeSpy to create those same visualizations. I believe that the success of a toolkit like this is strongly tied to its ease of use, and this aspect is not clear or prominently highlighted in the manuscript."

We have added the specification of the DECIDER visualization and a comprehensive README to a repository (https://github.com/HautaniemiLab/genomespy-paper-2024-spec), allowing readers to study how the visualization was built. The README explains how the visualization is organized and which key features were used. The downloadable repository serves as a starting point for users who wish to adapt the showcased visualization for their own data. We added a link to the repository to the paragraph that introduces the DECIDER visualization.

"Additionally, it would be interesting to more clearly highlight GenomeSpy's strengths compared to other approaches. By combining Rshiny and ggplot, it is indeed possible to create complex interactive data visualizations. Therefore, it would be necessary to more strongly emphasize what the other innovative aspects of GenomeSpy are, beyond GPU acceleration, compared to other approaches available today."

We have expanded the last paragraph under the "Results" heading, starting with "In addition to …". We now highlight GenomeSpy's data processing capabilities, which can be configured using the grammar. In addition, we also introduce the new parameterization support that enables advanced use cases, such as the configurable G score calculation shown in the updated Figure 7. Furthermore, we emphasize that GenomeSpy's architecture enables client-side data processing that necessitates no specialized server-side infrastructure, which, in contrast, would be necessary with visualizations based on Rshiny.

(An abstract example of the new parameterization feature: https://genomespy.app/docs/grammar/transform/formula/#using-with-parameters)

Reviewer 3

"Lavikka and coworkers present an interesting visualization framework and associated application for genomics visualization. The challenges outlined by the authors in finding appropriate visualization tools for large-scale genomics data were also experienced by this reviewer, and thus better and improved tools are

always welcome.
The manuscript is well laid out, presenting the key facts in a proper manner. The use of GPU rendering for graphs is an excellent move, and I expect to be extremely useful even for machines with lower-end GPUs. The code looks reasonably written and commented (being an application, this too is important for a review). I have also tested the examples, and indeed the software is very useful (the documentation should, however, point out that some issues regarding saving the canvas still exist)."

We agree that there is a need to save the canvas, and are planning to make it more straightforward to save the canvas contents to an image file: https://github.com/genome-spy/genome-spy/issues/246. As the documentation has currently no mention about saving the canvas, we have not discussed issues related to it in the documentation.

"One may argue that the use of JSON for the graph grammar can be awkward, but at the same time other file formats may be more problematic and/or require specialized parsers (which open yet another can of worms)."

In the revised version we clarified the shortcomings and opportunities of the JSON-based grammar in the last paragraph of discussion.

"As a minor suggestion, the authors may want to add some form of search to their documentation page."

We have now added a search functionality to the documentation website.

"There are is an open questions that the authors may want to answer: they explicitly mention GISTIC 1.0 for the G-score plots. Is there a specific reason why they chose 1.0? The 2.0 algorithm is far more robust and produces more reliable results."

GISTIC 2.0 introduced the Ziggurat Deconstruction algorithm to model the background mutation process. As in iterative algorithm, it cannot be implemented easily using GenomeSpy’s dataflow model. Moreover, the formula in GISTIC 1.0 is much faster to compute than the more complex 2.0 algorithm, which is preferred in an interactive application. We have updated the methods section with a brief rationale for the choice of GISTIC 1.0’s G score.
