# Supplementary material for: Deciphering cancer genomes with GenomeSpy: a grammar-based visualization toolkit
Source: Gigascience. 2024 Aug 5;13:giae040. doi: 10.1093/gigascience/giae040 (PMC11299109; doi:10.1093/gigascience/giae040)
Supplement: giae040_Response_to_Reviewer_Comments_Revision_1 [file giae040_response_to_reviewer_comments_revision_1.pdf]

Author's Response To Reviewer Comments

We have made the following changes to the manuscript:
- Added RRIDs to those software tools that have a record in SciCrunch
- Replaced URLs in figure captions with references that are cited in the captions.
- Also added Refs to some URLs that lacked it.
- Replaced Zenodo archives of the GenomeSpy toolkit and SegmentModel Spy with Software Heritage Archives, which are cited as suggested by Chris Armit.
- Also added the inadvertently omitted custom ASCAT implementation to Software Heritage Archive and cited it in the Methods and Data Availability sections.
- Changed the license of https://doi.org/10.5281/zenodo.11121377 to CC0 as required by GigaScience
- Updated the Funding section
- Fixed the format of references. DOIs are now URLs. Some referenced lacked the DOI altogether. Fixed those as well.

I have also sent the ORCIDs of all authors to Nafisa Qazi as an xlsx file.
