# Supplementary material for: Deciphering cancer genomes with GenomeSpy: a grammar-based visualization toolkit
Source: Gigascience. 2024 Aug 5;13:giae040. doi: 10.1093/gigascience/giae040 (PMC11299109; doi:10.1093/gigascience/giae040)
Supplement: giae040_Reviewer_1_Report_Original_Submission — Andrea Sboner, PhD -- 2/15/2024 Reviewed [file giae040_reviewer_1_report_original_submission.pdf]

Reviewer Report

Title: Deciphering Cancer Genomes with GenomeSpy: A Grammar-Based Visualization Toolkit

Version: Original Submission **Date:** 2/15/2024

Reviewer name: Andrea Sboner, PhD

Reviewer Comments to Author:

In this manuscript, the authors present Genome Spy, a visualization toolkit geared toward the rapid and interactive exploration of genomic features. They demonstrate how this tool can help investigators explore a large cohort of 753 ovarian cancers sequenced by whole-genome sequencing (WGS). By using the tool, they were able to identify outliers in the dataset and refine their diagnosis. The tool is inspired by Vega-lite, a high-level grammar for interactive graphics, and extends it for genomic applications.The manuscript is clearly written, and the authors provide links to the applications itself, tutorials and examples. I want to commend them for doing this. This is a tool that would nicely complement others and has a specific advantage of using high-performance GPUs that are now common in modern computers.The only concern that I have is about a couple of claims that may not be fully supported by the data provided:1. Claim: users can implement new visualization designs easily. While the grammar certainly enables the users to define new designs, I do not think that this is necessarily easy, as the authors themselves recognize in the discussion section when they suggest providing templates to reduce the learning curve. Indeed, the example in Figure 2 is still quite verbose and would need some time for anyone to understand the syntax and the style. The playground web application facilitates testing it, though.2. Claim: the grammar-based approach allows to be mixed and matched. I did not find any specific example of how to do this. It would have been quite interesting to see the intersection between the DNA representation of structural variants and RNA-seq data (if this is what it means as "mix and match")

Level of Interest

Please indicate how interesting you found the manuscript: Choose an item.

Quality of Written English

Please indicate the quality of language in the manuscript: Choose an item.

Declaration of Competing Interests

Please complete a declaration of competing interests, considering the following questions:

- Have you in the past five years received reimbursements, fees, funding, or salary from an organisation that may in any way gain or lose financially from the publication of this manuscript, either now or in the future?

- Do you hold any stocks or shares in an organisation that may in any way gain or lose financially from the publication of this manuscript, either now or in the future?
- Do you hold or are you currently applying for any patents relating to the content of the manuscript?
- Have you received reimbursements, fees, funding, or salary from an organization that holds or has applied for patents relating to the content of the manuscript?
- Do you have any other financial competing interests?
- Do you have any non-financial competing interests in relation to this paper?

If you can answer no to all of the above, write 'I declare that I have no competing interests' below. If your reply is yes to any, please give details below.

None

I agree to the open peer review policy of the journal. I understand that my name will be included on my report to the authors and, if the manuscript is accepted for publication, my named report including any attachments I upload will be posted on the website along with the authors' responses. I agree for my report to be made available under an Open Access Creative Commons CC-BY license (http://creativecommons.org/licenses/by/4.0/). I understand that any comments which I do not wish to be included in my named report can be included as confidential comments to the editors, which will not be published.

Choose an item.

To further support our reviewers, we have joined with Publons, where you can gain additional credit to further highlight your hard work (see: https://publons.com/journal/530/gigascience). On publication of this paper, your review will be automatically added to Publons, you can then choose whether or not to claim your Publons credit. I understand this statement.

Yes Choose an item.
