# Supplementary material for: Deciphering cancer genomes with GenomeSpy: a grammar-based visualization toolkit
Source: Gigascience. 2024 Aug 5;13:giae040. doi: 10.1093/gigascience/giae040 (PMC11299109; doi:10.1093/gigascience/giae040)
Supplement: giae040_Reviewer_3_Report_Original_Submission — Luca Beltrame, Ph.D. -- 3/11/2024 Reviewed [file giae040_reviewer_3_report_original_submission.pdf]

Reviewer Report

Title: Deciphering Cancer Genomes with GenomeSpy: A Grammar-Based Visualization Toolkit

Version: Original Submission **Date:** 3/11/2024

Reviewer name: Luca Beltrame, Ph.D.

Reviewer Comments to Author:

Lavikka and coworkers present an interesting visualization framework and associated application for genomics visualization. The challenges outlined by the authors in finding appropriate visualization tools for large-scale genomics data were also experienced by this reviewer, and thus better and improved tools are always welcome.The manuscript is well laid out, presenting the key facts in a proper manner. The use of GPU rendering for graphs is an excellent move, and I expect to be extremely useful even for machines with lower-end GPUs. The code looks reasonably written and commented (being an application, this too is important for a review). I have also tested the examples, and indeed the software is very useful (the documentation should, however, point out that some issues regarding saving the canvas still exist). One may argue that the use of JSON for the graph grammar can be awkward, but at the same time other file formats may be more problematic and/or require specialized parsers (which open yet another can of worms).Documentation is also logically organized. As a minor suggestion, the authors may want to add some form of search to their documentation page.There are is an open questions that the authors may want to answer: they explicitly mention GISTIC 1.0 for the G-score plots. Is there a specific reason why they chose 1.0? The 2.0 algorithm is far more robust and produces more reliable results.

Level of Interest

Please indicate how interesting you found the manuscript: Choose an item.

Quality of Written English

Please indicate the quality of language in the manuscript: Choose an item.

Declaration of Competing Interests

Please complete a declaration of competing interests, considering the following questions:

- Have you in the past five years received reimbursements, fees, funding, or salary from an organisation that may in any way gain or lose financially from the publication of this manuscript, either now or in the future?
- Do you hold any stocks or shares in an organisation that may in any way gain or lose financially from the publication of this manuscript, either now or in the future?

- Do you hold or are you currently applying for any patents relating to the content of the manuscript?
- Have you received reimbursements, fees, funding, or salary from an organization that holds or has applied for patents relating to the content of the manuscript?
- Do you have any other financial competing interests?
- Do you have any non-financial competing interests in relation to this paper?

If you can answer no to all of the above, write 'I declare that I have no competing interests' below. If your reply is yes to any, please give details below.

I declare that I have no competing interests.

I agree to the open peer review policy of the journal. I understand that my name will be included on my report to the authors and, if the manuscript is accepted for publication, my named report including any attachments I upload will be posted on the website along with the authors' responses. I agree for my report to be made available under an Open Access Creative Commons CC-BY license (http://creativecommons.org/licenses/by/4.0/). I understand that any comments which I do not wish to be included in my named report can be included as confidential comments to the editors, which will not be published.

Choose an item.

To further support our reviewers, we have joined with Publons, where you can gain additional credit to further highlight your hard work (see: https://publons.com/journal/530/gigascience). On publication of this paper, your review will be automatically added to Publons, you can then choose whether or not to claim your Publons credit. I understand this statement.

Yes Choose an item.
